# Preoperative gait patterns and BMI are associated with tibial component migration

**DOI:** 10.3109/17453674.2010.501741

**Published:** 2010-07-16

**Authors:** Janie L Astephen Wilson, David AJ Wilson, Michael J Dunbar, Kevin J Deluzio

**Affiliations:** ^1^School of Biomedical Engineering; ^2^Department of Surgery, Division of Orthopaedics, Dalhousie University, Halifax, Nova Scotia; ^3^Department of Mechanical and Materials Engineering, Queen's University, Kingston, OntarioCanada

## Abstract

**Background and purpose:**

There is no standard for patient triage in total knee arthroplasty (TKA) based on joint functional characteristics. This is largely due to the lack of objective postoperative measurement of success in TKA in terms of function and longevity, and the lack of knowledge of preoperative metrics that influence outcome. We examined the association between the preoperative mechanical environment of the patients knee joint during gait and the post-TKA stability of the tibial component as measured with radiostereometric analysis (RSA).

**Methods:**

37 subjects were recruited out of a larger randomized RSA trial. 3-dimensional gait analysis was performed in the preoperative week. Longitudinal RSA data were gathered postoperatively at 6 months and 1 year.

**Results:**

We found a statistically significant association between the pattern of the knee adduction moment during gait preoperatively and the total migration of the implant at 6 months postoperatively. A substantial proportion of the variability in the total postoperative tibial component migration (R^2^ = 0.45) was explained by a combination of implant type, preoperative knee joint loading patterns during gait, and body mass index at 6 months postoperatively. The relationships did not remain statistically significant at 1 year postoperatively.

**Interpretation:**

Our findings support the hypothesis that preoperative functional characteristics of patients, and particularly joint loading patterns during activities of daily living, are important for outcome in TKA. This represents a first step in the development of predictive models of objective TKA outcome based on preoperative patient characteristics, which may lead to better treatment strategies.

ClinicalTrials.gov (NCT00405379)

## Introduction

Total knee arthroplasty (TKA) for end-stage osteoarthritis (OA) is highly effective according to the commonly employed self-report measures of pain and function, and patients are generally very satisfied with the results of the procedure (Robertsson and Dunbar 2001). Aseptic loosening of the tibial component is the leading cause of revision surgery (CIHI 2006). Subjective outcome questionnaires tend to reflect the reduction of symptoms associated with TKA and not the functional state of the joint. They are therefore insensitive for detection of the early manifestations of aseptic loosening, and often problems associated with implant design or surgical technique do not become apparent for many years after surgery.

Radiostereometric analysis (RSA) is a highly accurate technique for measurement of the relative movement between segments, and it is beginning to be used in orthopedic practice as an objective measure of post-TKA implant stability (Albrektsson et al. 1992, Ryd et al. 1995, Dunbar et al. 2009). RSA has been shown to measure movement of the tibial component of a TKA within the native surrounding bone with high accuracy (Ryd 1986). Early migration of implant components, as measured with RSA, has also been shown to be predictive of later loosening and thus failure of the implant (Ryd et al. 1995). RSA is therefore a sensitive and accurate tool that can be used early on postoperatively to predict long-term outcomes.

Despite these advances in using RSA as an objective measure of postoperative outcome, the question still remaining is why some implants remain stable for years postoperatively while others migrate continuously and must eventually be revised. While patient selection can affect surgical outcome, little is known about which preoperative metrics are predictive of long-term implant fixation. Intuitively, one could predict that the mechanical environment of the knee joint would influence the mechanical fixation of the implant, and walking is the best model to measure this effect as it is the most common and repetitive dynamic human task (Guccione 1994). Yet, there have been very few studies dealing with the association between dynamic joint loading during gait and postoperative implant migration. Hilding et al. (1995, 1999) found that patients whose implants were continuously migrating in RSA follow-up examinations walked with higher peak knee flexion moments during gait both before and after TKA surgery. However, they did not find any association between implant migration and the knee adduction moment, the measure of gait most commonly associated with severity of knee OA, progression, and surgical outcome (Prodromos et al. 1985, Hurwitz et al. 2002, Miyazaki et al. 2002). This may be due to the analysis method employed in the study. The gait variables analyzed were peak values extracted from the waveform pattern of the gait measures over the gait cycle. Recent work by our group has shown that the time pattern of the knee adduction over the gait cycle may be a more significant indicator of disease severity than the commonly analyzed peak value of the moment during gait (Landry et al. 2007, Astephen et al. 2008a).

Body mass is an additional factor that influences joint loading, and it is highly associated with OA of the knee. Obesity is one of the most cited risk factors for development of knee OA (Felson et al. 1988a), and more than 80% of Canadian adults treated with TKA are either overweight or obese (CIHI 2006). Understanding how body mass relates to implant loading and consequent long-term outcome is particularly important in orthopedic triage and surgical planning and design. The primary objective of this study was to investigate the associations between the preoperative knee adduction moment during gait and BMI and early implant migration as measured with RSA at 6 months postoperatively on the other. A secondary objective was to examine the multivariate association between preoperative joint loading and BMI on the one hand and post-TKA implant migration on the other.

## Patients and methods

This study involved a subset of patients (40) who took part in a larger randomized controlled trial (n = 70). The study was registered at ClinicalTrials.gov (NCT00405379) and was performed in accordance with the Declaration of Helsinki. Patients with end-stage primary knee OA were recruited from the TKA waiting list at the Orthopaedic Clinic of Halifax Infirmary. Patients were randomized to receive the Nexgen LPS Trabecular Metal tibial monoblock component (n = 37) (Zimmer, Warsaw, IN) or the cemented NexGen Option Stemmed tibial component (n = 33) (also Zimmer). The duration of t,he study was 2 years from the time of surgery, and the primary endpoint was RSA migration data at 2 years.

Surgery was performed by 4 experienced consultant knee surgeons using a standardized protocol: posterior cruciate ligament resection, patellar resurfacing with a cemented inlay component, cementing of the femoral component, and RSA marker placement of 0.8-mm beads. 4–6 tantalum markers were placed around the periphery of the polyethylene component and 8–20 tantalum markers were inserted into the proximal tibia. The postoperative protocol was standardized with the use of continuous passive motion as tolerated, and patients were allowed full weight bearing immediately after surgery. No drains were used.

**Figure 1.**
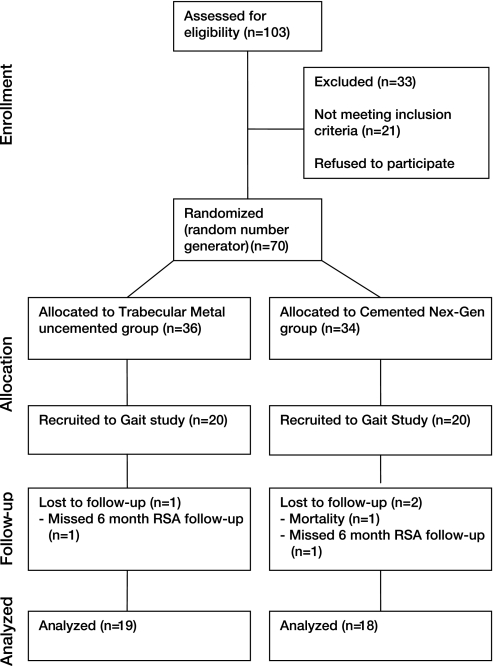
CONSORT flow diagram.

Of these 70 patients, 40 of them (20 Trabecular Metal, 20 Cemented NexGen) were recruited to undergo 3-dimensional gait analysis testing within a week before surgery (Figure 1). Patients were included in the gait analysis part of the study if they were able to walk along a 6-m walkway without a walking aid, and they were excluded if they had any neuromuscular disease, cardiovascular disorders, or had had lower limb surgery (with the exception of exploratory arthroscopy, lavage of the knee joint, or partial menisectomy at least 1 year before entry into the study). Informed consent was obtained from all participants in accordance with the Ethics Review Board of the Capital District Health Authority.

### Gait analysis

During the gait trials, 3-dimensional movement of the affected lower limb and external ground reaction forces were recorded with a synchronized Optotrak 3020 motion capture (Northern Digital Inc., Waterloo, ON) and force platform system (AMTI, Watertown, MA). Walking speed was monitored using infrared timing gates, and participants were required to complete 5 trials within 5% of their self-selected walking velocity.

Triads with 3 infrared light-emitting diodes were placed on the pelvis, thigh, leg, and foot segments of the affected leg. Individual diodes were placed on the greater trochanter, lateral epicondyle, lateral malleolus, and shoulder. These and 8 virtual markers (right and left anterior superior iliac spines, medial epicondyle, tibial tuberosity, fibular head, medial malleolus, second metatarsal, and heel) were used to define anatomical coordinate systems in each of the lower extremity segments (Landry et al. 2007). 3-dimensional joint angles at the knee were calculated over a complete gait cycle using a least-squares optimization routine (Challis 1995). Net joint moments at the knee were calculated with an inverse dynamics procedure (Braune and Fischer 1987), normalized to body mass and the anatomical joint coordinate system described by Grood and Suntay (1983). All joint angles and moments were time-normalized and defined with 101 data points, 1 for each percentage of the gait cycle.

### Radiostereometric analysis

Within 4 days of surgery and at 6 and 12 months postoperatively, the knee was placed within a biplanar calibration box (Tilly Medical Products AB, Lund, Sweden), and simultaneous digital stereo radiographs were taken with the tubes oriented orthogonally. RSA analysis was performed with MB-RSA (MEDIS, Leiden). RSA results at 6 months and 1 year were reported as maximum total point motion (MTPM) and 6 degrees-of-freedom translations and rotations. MTPM is the 3-dimensional (3D) vector magnitude of the marker that has exhibited the most migration between examinations. The RSA calculations gave the relative motion of the rigid body defined by the beads in the prostheses, with respect to the rigid body defined by the beads in the tibia in 3 dimensions. Rigid body rotations of the prosthesis were calculated about a coordinate system centered at the volumetric center of the implant with axes aligned to the anatomical directions. All translations and rotations were calculated for a right-hand coordinate system with the signs corrected to comply with the standards presented by Valstar et al. (2005).

MTPM for each participant was calculated using fictive markers, a set of virtual points defined in the rigid body of the implant. Fictive markers are used to standardize the MTPM calculations in cases where the prosthesis bead placement is not uniform across all subjects (Nilsson et al. 1999, Valstar et al. 2005). 6 fictive markers were placed at the periphery of the implant volume. The rotations and translations of the rigid body defined by the tantalum beads in the polyethylene about the volumetric centroid of the prosthesis were then applied to the fictive points to calculate the MTPM for each follow-up. The limit of rigid body fitting was a maximum of 0.2 mm for the tibial segment and 0.2 mm for the prosthesis segment. In any cases where the rigid body errors exceeded the threshold due to a loose bead, that bead was removed from the analysis. The condition number did not exceed 40 at any follow-up examination, indicating that there was adequate distribution of beads in the rigid body (Valstar et al. 2005).

The accuracy of the RSA system was assessed with a standard phantom study protocol. Accuracy was represented as half of the average width of the 95% prediction interval in a regression analysis of true and measured translations of a phantom. Precision was evaluated with double-examination analysis (Valstar et al. 2005), and represented as the 95% confidence interval of the measurements from 11 double clinical examinations.

### Statistics

The 3-dimensional angles and moments at the knee joint for the 5 gait trials were averaged to create ensemble average profiles for each measure and subject. Each measure was represented as a waveform (101 data points each to represent one complete gait cycle, from 0 to 100%). It was hypothesized that knee joint loading patterns would be more associated with implant migration than movement patterns. Thus, the 3 dimensions of net knee joint moments (3 measurements in total) were included in the analysis.

Principal component analysis (PCA), a multivariate statistical analysis technique, was applied to each gait waveform measure (X = 101 × 37; see below) separately to extract the pattern of variability of each of the measures over the gait cycle. PCA has been shown to be an effective tool in the reduction and interpretation of gait waveform data (Deluzio and Astephen 2007), extracting the most important patterns of variability within subject waveforms called principal components (PCs). Each gait waveform is presented by a matrix, X, which is a 101 (stride normalized data points) by 37 (number of subjects) matrix of a knee moment waveform (knee adduction, flexion, or rotation moment). PCs are the eigenvectors of the covariance matrix of X (101-dimensional), and capture the different principal patterns of variability in the original waveform data. The projection of each subjects waveform data onto a PC results in a set of discrete PC scores with which to compare the gait waveform patterns between subjects. Differences in PC scores for a given gait measure therefore reflect differences in one of the principal patterns within the waveform data. Most of the variability within the original data (> 90%) is generally captured in the first few principal components extracted, so the first 3 PCs of each of the 3 dimensions of knee moments were extracted for further analysis (3 PCs × 3 gait measures = 9 variables in total). PCs were interpreted by examining the pattern of the eigenvector over the gait cycle, as well as by examining subject waveforms in the fifth and ninety-fifth percentiles of the PC scores (Deluzio and Astephen 2007).

Due to the non-parametric distribution of the MTPM data, a log transformation was applied to the data for further statistical modeling. Pearson correlations were used to examine the relationships between the first principal component (PC) score for each gait variable (3 dimensions of knee joint moments) and BMI with log MTPM, and the association between the knee adduction moment PC1 and varus tilt of the implant postoperatively. A Bonferroni-corrected level of significance of p = 0.01 was used to account for 5 statistical comparisons (p = 0.05/5 = 0.01; using p = 0.05 for each individual test). 2 multiple linear regression models were defined with log MTPM (at 6 months and 1 year) as the dependent variable, and implant type, BMI, and the first principal component scores of the knee adduction moment and the knee flexion moment as the independent variables. These particular PC scores were chosen for the multivariate model because PC1 of the knee adduction moment represents the overall magnitude of the varus/valgus moment on the knee joint during the stance phase of walking, which is the gait variable most commonly used as a metric of frontal plane loading and most commonly associated with progression of knee osteoarthritis (Hurwitz et al. 2002, Mundermann et al. 2004, Landry et al. 2007, Astephen et al. 2008a). PC1 of the knee flexion moment also represents the overall magnitude of the moment during stance, and this measure has been shown in previous studies to be associated with implant migration (Hilding et al. 1996). Factors were kept in the multivariate model if they contributed in a statistically significant way to the explanation of log MTPM, given the other factors in the model (p < 0.01). Only those PCs that were statistically significantly correlated to RSA data or included in the multivariate models were interpreted. Multiple regression model diagnostics included residual analyses, multicollinearity analyses, and inferential analyses.

## Results

3 subjects were lost to follow-up at 6 months postoperatively, leaving 19 TM and 18 NG patients. 1 patient died, and 2 missed RSA follow-up examinations. 4 additional subjects were lost to follow-up at 1 year, leaving 17 TM and 16 NG patients for the 1-year RSA results. The 4 additional patients who were lost at 1 year missed their 1-year follow-up RSA appointments. The TM and NG groups were similar in terms of age, BMI, and gait speed (Table 1). The mean MTPM for the total group was 0.62 mm at 6 months and 0.63 mm at 1 year (Table 2). There was no statistically significant difference in MTPM between the TM and NG groups at either 6 months (p = 0.2) or 1 year (p = 0.1). There was no statistically significant difference in MTPM between the 6-month follow-up and the 1-year follow-up (p = 0.9). There was a statistically significant difference in translation in the y-direction (proximal-distal) between the groups at 6 months and 1 year, with the TM group showing more distal migration at both follow-up times (p < 0.001). None of the subjects included in this analysis were considered to have more continuous migration (with migration at 12–24 months all < 0.2 mm) (Ryd et al. 1995); the longitudinal RSA results of these subjects have been reported previously (Dunber et al. 2009).

**Table 1.** Subject demographics. There were no statistically significant differences in age, BMI, or gait speed between the TM and NG groups

	Total group		TM group		NG group	
	mean	range	mean	range	mean	range
Age (years)	64.4	42–82	64.9	42–77	63.8	46–82
BMI (kg/m^2^)	33.0	22.0–42.6	31.8	22.0–42.6	34.3	22.9–42.4
Gait speed (m/sec)	0.93	0.38–1.39	0.91	0.57–1.31	0.94	0.38–1.39

**Table 2.** RSA results

Direction	Total group	TM group	NG group
6-month results			
MTPM (mm)	0.62 (0.06–2.53)	0.79 (-0.06–2.53)	0.44 (0.10–1.64)
Log MTPM	-0.39 (-1.21–0.40)	-0.30 (-1.21–0.40)	-0.48 (-0.99–0.21)
Tx (mm)	0.00 (-0.22–0.46)	0.00 (-0.12 –0.25)	0.00 (-0.22–0.46)
Ty (mm) **^a^**	-0.18 (-1.00–0.16)	-0.36 (-1.00 to -0.02)	0.01 (-0.15–0.16)
Tz (mm)	0.04 (-0.64–0.85)	0.03 (-0.29–0.30)	0.06 (-0.64–0.85)
Rx (°)	-0.13 (-1.34–1.19)	-0.30 (-1.34–1.19)	0.05 (-0.64–0.85)
Ry (°)	-0.04 (-1.75–1.36)	0.10 (-0.40–1.36)	-0.19 (-1.75–0.42)
Rz (°)	0.14 (-0.58–1.38)	0.24 (-0.58–1.38)	0.02 (-0.35–0.38)
1-year results			
MTPM (mm)	0.63 (0.11–2.87)	0.66 (0.17 –2.87)	0.60 (0.11–1.70)
Log MTPM	-0.34. (-0.96–0.46)	-0.29 (-0.76–0.46)	-0.40 (-0.96–0.23)
Tx (mm)	0.00 (-0.26–0.49)	0.00 (-0.17–0.18)	0.00 (-0.26–0.49)
Ty (mm) **^a^**	-0.17 (-1.15–0.38)	-0.33 (-1.15 to -0.06)	0.01 (-0.11–0.38)
Tz (mm)	0.07 (-0.21–0.56)	0.02 (-0.21–0.27)	0.11 (-0.09–0.56)
Rx (°)	-0.15 (-1.70–1.43)	-0.31 (-1.70–1.43)	0.01 (-0.63–0.78)
Ry (°)	0.01 (-1.11–1.46)	0.10 (-0.40–1.46)	-0.08 (-1.11–0.61)
Rz (°)	0.09 (-0.44–1.27)	0.17 (-0.43–1.27)	0.00 (-0.39–0.29)

**^a^** denotes significant difference (p < 0.05) between TM and NG group.

Results of the phantom study showed that the accuracy of the RSA system was 0.02 mm, 0.02 mm, 0.06 mm, and 0.03 mm for the x-, y-, and z-directions and MTPM, respectively. The precision of the RSA system was 0.07 mm, 0.07 mm, 0.11 mm, 0.16°, 0.15°, 0.12°, and 0.10 mm for the x-, y-, and z-translations, the x-, y-, and z- rotations, and MTPM, respectively.

### Principal component and RSA correlation analysis

**Figure 2.**
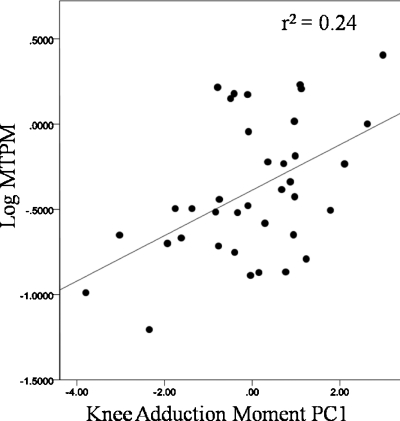
Knee adduction/abduction moment PC1 and 6-month log MTPM scatter plot. A statistically significant correlation was found between the preoperative pattern of the knee adduction moment (PC1) and the log transform of postoperative total implant migration (MTPM) at 6 months postoperatively.

A statistically significant correlation was found between the first principal component (PC1) extracted from the knee adduction moments and log MTPM at 6 months (r^2^ = 0.24 (0.04–0.50), p = 0.002) (Figure 2). PC1 of the knee adduction moment was also associated with Rz at 6 months (r^2^ = 0.15 (0.005–0.41), p = 0.02), which represented the varus/valgus tilt of the implant within the tibia, but this association was not statistically significant. BMI was associated with log MTPM at 6 months (r^2^ = 0.14 (0.004–0.40), p = 0.02), but the correlation was not statistically significant with the Bonferroni correction. However, because the p-value was small, this factor was included in the multivariate model. There were no statistically significant correlations between any individual factors and log MTPM at 1 year. The correlation between PC1 of the knee adduction moment and log MTPM was not as strong at 1 year and was no longer significant (r^2^ = 0.16 (0.01–0.43), p = 0.02).

**Figure 3.**
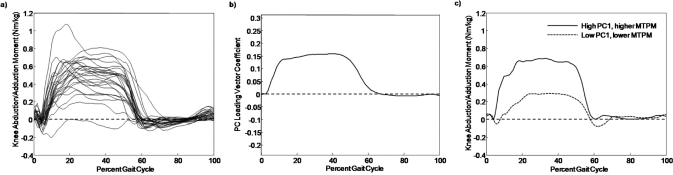
Knee adduction/abduction moment PC1. a. The original knee adduction moment waveforms for 1 complete gait cycle for all subjects are shown. Positive moments are adduction moments. b. The PC1 loading vector for the knee adduction moments captures the variability in the overall magnitudes of the moments during the stance phase of the gait cycle. c. Representative subject knee adduction moment waveforms are shown to demonstrate the difference between a high (ninety-fifth percentile) PC1 score and a low (fifth percentile) PC1 score. Higher PC1 scores represented higher stance phase knee adduction moments and were significantly correlated with rank MTPM and significant and positive in the multivariate regression model for log MTPM.

The original knee adduction moment waveforms for all patients preoperatively during gait are shown in Figure 3a. PC1 of the knee adduction moment explained 85% of the variability in the original knee adduction moment waveforms and was interpreted as the overall magnitude of the moment during the stance phase of the gait cycle, because the loading vector was constant and positive over the majority of the stance phase (Figure 3b). Example knee adduction moment waveforms with high and low PC1 scores (ninety-fifth and fifth percentiles) illustrate this interpretation (Figure 3c). Higher PC1 scores, and therefore higher magnitudes of knee adduction moment during stance preoperatively, were associated with higher total motion of the tibial component (MTPM) and greater varus tilt relative to the surrounding bone at 6 months postoperatively.

**Figure 4.**
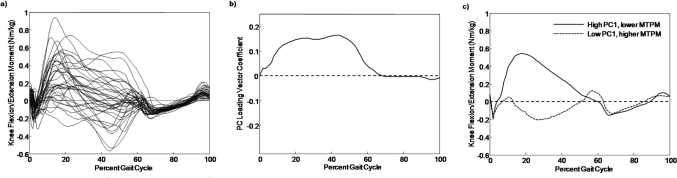
Knee flexion/extension moment PC1. a. The original knee flexion moment waveforms for one complete gait cycle for all subjects are shown. Positive moments are flexion moments. b. The PC1 loading vector for the knee flexion moments captures the variability in the overall magnitudes of the moments during the stance phase of the gait cycle. c. Representative subject knee flexion moment waveforms are shown to demonstrate the difference between a high (ninety-fifth percentile) PC1 score and a low (fifth percentile) PC1 score. Higher PC1 scores were associated with overall greater knee flexion moments during stance. Lower PC1 scores were associated with a more [word missing?] extension moment pattern during stance and lower scores were associated with higher MTPM in the multivariate regression model.

The results of the multiple linear regression analysis for MTPM at 6 months showed that log MTPM was well explained by the combination of implant group, PC1 of the knee adduction moment, BMI, and PC1 of the knee flexion moment (R2 = 0.45 (0.19–0.67), R^2^ adjusted = 0.39, p = 0.001). All variables included were statistically significant in the model (p < 0.10). For the multivariate model with log MTPM at 1 year using the same 4 factors, only PC1 of the knee adduction moment and BMI remained significant in the model (implant group: p = 0.1; BMI: p = 0.04; PC1 knee adduction moment: p = 0.09; PC1 knee flexion moment: p = 0.33). A second multivariate model with only PC1 of the knee adduction moment and BMI showed that BMI was no longer significantly associated? in the multivariate model with PC1 of the knee adduction moment (BMI: p = 0.13; PC1 knee adduction moment: p = 0.04). The final model for log MTPM at 1 year therefore only included PC1 of the knee adduction moment, and the strength of the relationship was reduced (R^2^ = 0.16 (0.01–0.43), R^2^ adjusted = 0.14, p = 0.02). Residual, influence, and multicolinearity tests indicated that there was no violation of model assumptions, no multicolinearity problems, and the residuals followed a normal distribution. In the multivariate models, higher knee adduction moments during stance were again associated with higher MTPM. PC1 of the knee flexion moment explained 74% of the variability in the original knee flexion moment waveforms. It was interpreted as the overall magnitude of the moment during the stance phase of the gait cycle (Figure 4). Higher PC1 scores were associated with higher flexion moment magnitudes during stance. In the multivariate models, MTPM was associated with lower knee flexion moment PC1 scores or lower magnitudes of flexion moment during stance. Also in the multivariate model, having an uncemented TM implant was also associated with higher migration at 6 months and 1 year than having a cemented NG. Thus, in combination, higher knee adduction moments and lower knee flexion moments during stance, an uncemented implant and higher BMI preoperatively were associated with higher MTPM of the implant within the tibia at 6 months postoperatively. The results of the regression analyses are summarized in Table 3.

**Table 3.** Multiple linear regression results for log MTPM

Variable	Coefficient	Coefficient CI	p-value
6-month results			
Constant	–1.49	(–2.22 to 0.76)	0.001
Knee adduction moment PC1	0.10	(0.02 to 0.17)	0.01
Knee flexion moment PC1	0.07	(–0.01 to 0.15)	0.08
BMI	0.03	(0.01 to 0.05)	0.006
Implant group	0.21	(–0.01 to 0.43)	0.06
1-year results			
Constant	–0.36	(–0.47 to –0.24)	< 0.001
Knee adduction moment PC1	0.09	(0.15 to 0.17)	0.02

## Discussion

Our findings support the hypothesis that preoperative knee joint loading patterns during gait are associated with the postoperative migration of the tibial component of a TKA implant within the surrounding bone. The dynamic knee adduction moment during gait is the mechanical variable most associated with knee osteoarthritis both pre- and postoperatively (Prodromos et al. 1985, Miyazaki et al. 2002, Hurwitz et al. 2002). While the knee adduction moment has been identified as predictive of the outcome of high tibial osteotomy knee surgery (Prodromos et al. 1985), our study is the first to indicate that there is a statistical association between the preoperative knee adduction moment and an objective outcome of TKR surgery.

We found that there was a correlation between the overall magnitude of the knee adduction moment during stance preoperatively and postoperative migration of the tibial component within the surrounding bone. The only other authors to examine the association between preoperative gait patterns and postoperative implant migration did not report any relationship between the knee adduction moment and excessive postoperative migration (Hilding et al. 1995). This previous study examined the differences between a group whose implant stabilized within the body and a group “at risk” of early loosening. Our study instead investigated the associations between preoperative gait patterns and the continuum of migration values postoperatively rather than differences in dichotomous subject groups. We feel that this information may be associated with a potential mechanism of loosening rather than being diagnostic of loosening. Additionally, we employed principal component analysis, which extracts and analyses variability in the continuous pattern of gait measures as they change throughout the gait cycle, rather than the commonly employed subjective extraction of parameters from the waveform. This analysis technique is capable of identifying shape changes in gait measures that may not be reflected in peak values alone, and that may be important in understanding differences between subject gait patterns (Deluzio and Astephen 2007). PCA may have provided the additional sensitivity required to identify the potential importance of the knee adduction moment. The added usefulness of PCA in understanding gait patterns associated with knee osteoarthritis has been demonstrated in a number of previous studies (Astephen and Deluzio 2005, Deluzio and Astephen 2007, Landry et al. 2007, Astephen et al. 2008b).

Hilding et al. (1999) observed that a group of TKA patients found to be “at risk” of early aseptic loosening walked with higher knee flexion moments (mean and peak value) during gait both pre- and postoperatively. Somewhat in contrast, we found higher levels of migration to be associated with a pattern of a more constant extension moment during the stance phase of gait. Walking with a constant external knee extension moment would reflect a more stiff-kneed walking pattern (i.e. less range of flexion/extension angle), which would not have the characteristic load acceptance phase and associated mid-stance knee flexion angle during early stance of an asymptomatic pattern. This pattern would therefore also be associated with greater impact loading during early stance due to the lack of shock absorbency, which may have a detrimental effect on the mechanical stability of the implant. The difference between our results and those of Hilding et al. (1995, 1999) may again be attributed to the differences in our study populations and analysis techniques. Our results reflect the multivariate association of gait measures with the continuum of migration early in the postoperative period.

Knee OA is a complex, multifactorial disease process that involves the interactions between multiple factors, and combinations of mechanical variables have been shown to be more important in describing disease severity than individual factors alone (Astephen and Deluzio 2005, Astephen et al. 2008b). It is therefore likely that multivariate combinations of preoperative mechanical factors would be more important to surgical outcome than any individual factor alone. As expected, our results showed that postoperative implant migration was better explained by a combination of preoperative biomechanical variables than the knee adduction moment alone. This combination associates differences in the loading pattern within the joint in 2 anatomical planes (flexion/extension, abduction/adduction) and over the stance phase of the gait cycle, with higher postoperative migration.

Most patients who undergo total knee arthroplasty in Canada are overweight or obese (CIHI 2006). Obesity is one of the most cited risk factors for knee OA (Felson and Chaisson 1997, Manek et al. 2003). It has been associated with an increased risk of radiographic and symptomatic OA (Felson et al. 1988b, Spector et al. 1994) and accelerated disease progression (Schouten et al. 1992). Apart from the additive affect of increased body weight to joint loading during gait, obesity can cause immobility and movement adaptations that may adversely affect the loading environment within the joint, increasing the potential for injury (Wearing et al. 2006). It has been shown that obese individuals walk with increased total energy expenditure (Browning et al. 2006), and with increased magnitude and rate of ground reaction force. In our study, the combination of increased BMI and altered joint loading during gait preoperatively was associated with postoperative implant migration, which supports the notion that BMI is an important factor in patient triage and implant selection. In previous work on multivariate studies of knee OA, we have identified the added importance of BMI in combination with other gait characteristics (Astephen and Deluzio 2005, Astephen et al. 2008a). The results of this study further support the multivariate role of obesity in OA pathomechanics and surgical outcome, where the detrimental loading on the joint is dependent on the interaction between the static mass of the patient and the dynamic mechanical environment.

The most important conclusion from our work is the recognition of a multivariate statistical association between joint loading during gait and BMI with implant migration. While a larger sample size would be required to more definitively define the multivariate form of the relationship between MTPM and joint loading, this is a first step in our understanding of joint function and TKA outcome. The results of this study support the need for further investigation into the myriad of preoperative factors that can predict objective post-TKA outcome. The unexplained variability in postoperative implant migration may be accounted for by the differences in other patient characteristics not included in the current analysis such as bone density, sex, bone morphology, implant sizing and positioning, realignment of the joint intraoperatively, and other surgical factors.

The multivariate statistical association between preoperative knee joint loading and postoperative implant migration was stronger at 6 months postoperatively than at 1 year, although migration values were similar between the 2 time points. The knee adduction moment magnitude was the only statistically significant factor included in the regression model for MTPM at 1 year. It is intuitive that if the loading environment of the knee joint preoperatively would translate into the postoperative period, the relationship would weaken further into the postoperative period as biological mechanisms (i.e. bone ingrowth in uncemented implants. Also, because none of these implants migrated continuously, the predominant motion of the implant within the bone occurred early in the postoperative period (as shown in a previous study (Dunbar et al. 2009)), and so the association was more pronounced in this early preoperative period.

While the goal of this study was to examine the association between preoperative function and postoperative implant longevity, it is important to realize the functional adaptations that are provided by the implant post-surgically. Part of the standard operative procedure in TKA involves a correction of varus deformity to more neutral alignment. While some studies have associated static joint alignment and dynamic frontal plane joint loading (Olney et al. 1994, Hurwitz et al. 2002), others have not (Prodromos et al. 1985). This raises the question of whether metrics of dynamic (i.e. the knee adduction moment during gait) or static joint alignment should be used as surgical indicators in TKA. The findings of a related study by our group showed that the overall magnitude of the dynamic knee adduction moment during gait decreased from the preoperative to the postoperative state; however, the pattern of frontal plane loading during gait did not return to normal (Hatfield et al. 2010).

This study provides an indication that preoperative gait dynamics are associated with postoperative movement of the implant within the bone, but it was limited in terms of the number of variables investigated. While half of the variability in postoperative outcome is significant, future work should aim to capture and model all sources of variability, which include intraoperative and postoperative factors such as details of the surgical procedure, postoperative rehabilitation, bone density, etc. A second limitation of the study was sample size. While some statistically significant correlations have been identified, the study may have been underpowered to be able to detect statistically significant correlations with other metrics. Future multivariate analyses with more variables will require larger patient populations.

Providing objective information on implant survivorship based on preoperative metrics can have substantial implications for patient triage, implant design, and surgical technique. Such investigations are becoming increasingly important with the growing need for implant longevity and functionality, considering the expanding younger and more active patient demographic for TKR.
